# Correction: Youth focused life skills training and counselling services program–An inter-sectoral initiative in India: Program development and preliminary analysis of factors affecting life skills

**DOI:** 10.1371/journal.pone.0345082

**Published:** 2026-03-13

**Authors:** Gautham Melur Sukumar, Pradeep S. Banandur, Srividya Rudrapattana Nagaraja, Anusha B. Shenoy, Swati Shahane, Ravi G. Shankar, Arvind Anniappan Banavaram, Gananatha Shetty Yekkar, Shalini Rajneesh, Gururaj Gopalkrishna

The corresponding author’s email address is incorrect. Pradeep S. Banandur’s email address is: doctorpradeepbs@gmail.com.

In the Testing association between participant related independent factors and their level of life skills subsection of the Methodology, there is an error in the first sentence of the first paragraph. The correct sentence is: A total of 2037 participants are trained as on March 21 through 68 training programs.
