# Supplementary material for: Youth focused life skills training and counselling services program–An inter-sectoral initiative in India: Program development and preliminary analysis of factors affecting life skills
Source: PLoS One. 2023 Aug 24;18(8):e0284771. doi: 10.1371/journal.pone.0284771 (PMC10449178; doi:10.1371/journal.pone.0284771)
Supplement: S1 File — (DOCX) [file pone.0284771.s002.docx]

Protocol for Data Collection for the program Life skills training and counselling services program

## The change in different domains of the Life Skills training program will be assessed pre and post training using the finalized study instruments. The follow-up of study subjects will be for the duration of one year. The study subjects will be assessed prior to and immediately after the training program, at 3^rd^, 6^th^ and at 12^th^ month after the ‘Life Skills Training’ program. The participants participating in the ‘Life Skills training’ program will be in a staggered fashion and their follow-up period will start right after undergoing the training program. The data collection process for comparison group will be the same as the study participants.

## Electronic data collection system will be used for data collection. The field liaison officer and the Training Co-ordinator will participate in the data collection process. This will reduce the time lag from data collection to data entry while using a paper and pen format. In addition, this method will ensure paperless data collection. All data will be password protected and identified using unique identification number. Only the Principal Investigators of the study will have access to these data.

## Socio-demographic information will be obtained to adjust for confounding related to age, gender, experience and other correlates. A group of field interviewers will be blinded to the training status of participants of the study, and they will collect the data for the study. All assessments will be done on both the groups during the same time. Data collection procedures will be documented in a written protocol to ensure that data are collected in a standardized way.
