# Supplementary material for: Youth focused life skills training and counselling services program–An inter-sectoral initiative in India: Program development and preliminary analysis of factors affecting life skills
Source: PLoS One. 2023 Aug 24;18(8):e0284771. doi: 10.1371/journal.pone.0284771 (PMC10449178; doi:10.1371/journal.pone.0284771)
Supplement: Attachment — Submitted filename: Editor comments.docx [file pone.0284771.s003.docx]

Introduction:

1. Line 87: Authors mention tobacco use (35.6%), however the reference no. 5 does not have a link and the citation will not lead to appropriate reference. Pls verify.

Methodology

1. Page 7. Line 143 to 145: We finally reviewed 77 articles to gather information regarding different modules used for youth specific Life skills training and available tools to assess the effectiveness of such life skills training program.

Needs to be mentioned in results section

2. Table 1: Section 1, Interview information. The points are not clear, need to elaborate.
3. Pg 14. Line 216. The Data collection procedures documented in a written protocol can be provided as supplementary material for reference of readers.
4. Pg. 15, Line 222 -223. Rephrasing required.

Discussion:

1. Repetition can be avoided. Line 377 to 379 has been mentioned in earlier section also.
2. Pg 29. Line 440 to 449. It would be better to restrict the findings to present study.

References

1. Ensure links are working.
2. Ref 5, 7 and 22 needs to be as per the standard guidelines.
