# Supplementary material for: Youth focused life skills training and counselling services program–An inter-sectoral initiative in India: Program development and preliminary analysis of factors affecting life skills
Source: PLoS One. 2023 Aug 24;18(8):e0284771. doi: 10.1371/journal.pone.0284771 (PMC10449178; doi:10.1371/journal.pone.0284771)
Supplement: Attachment — Submitted filename: Response to Reviewers.docx [file pone.0284771.s004.docx]

To,

The Editor,

PLOS One Journal

Manuscript ID:

Dear Editor,

Sub: Response to Editor/Reviewer comments

At the outset, we would like to thank you for providing us your valuable inputs for the manuscript. We feel grateful to have received your valuable comments and suggestions which helped us in revising and improving our paper. We have tried our best to address all the reviews. We feel that this paper is now enriched, more clear and complete. Thank you once again. Please find our point-by-point response to all the comments below.

Journal Requirements:

When submitting your revision, we need you to address these additional requirements.

1. Please ensure that your manuscript meets PLOS ONE's style requirements, including those for file naming. The PLOS ONE style templates can be found at

<https://journals.plos.org/plosone/s/file?id=wjVg/PLOSOne_formatting_sample_main_body.pdf> and

<https://journals.plos.org/plosone/s/file?id=ba62/PLOSOne_formatting_sample_title_authors_affiliations.pdf>.

Response: Thank you, the template links were helpful. We have re-checked the PLOS ONE’s style requirements, including those for file naming according to the journal templates and made necessary changes.

2. Please provide additional details regarding participant consent. In the ethics statement in the Methods and online submission information, please ensure that you have specified what type you obtained (for instance, written or verbal, and if verbal, how it was documented and witnessed). If your study included minors, state whether you obtained consent from parents or guardians. If the need for consent was waived by the ethics committee, please include this information.

Once you have amended this/these statement(s) in the Methods section of the manuscript, please add the same text to the “Ethics Statement” field of the submission form (via “Edit Submission”).

For additional information about PLOS ONE ethical requirements for human subjects research, please refer to <http://journals.plos.org/plosone/s/submission-guidelines#loc-human-subjects-research>.

Response: We have added details about written informed consent obtained by participants in the methods section (Line: 186) as well as mentioned it under ethics statement (Line: 270) in the manuscript as well as made the changes in online submission information. Thank you.

3. We note that you have indicated that data from this study are available upon request. PLOS only allows data to be available upon request if there are legal or ethical restrictions on sharing data publicly. For more information on unacceptable data access restrictions, please see <http://journals.plos.org/plosone/s/data-availability#loc-unacceptable-data-access-restrictions>.

In your revised cover letter, please address the following prompts:

a) If there are ethical or legal restrictions on sharing a de-identified data set, please explain them in detail (e.g., data contain potentially sensitive information, data are owned by a third-party organization, etc.) and who has imposed them (e.g., an ethics committee). Please also provide contact information for a data access committee, ethics committee, or other institutional body to which data requests may be sent.

Response: Details provided in the cover letter.

b) If there are no restrictions, please upload the minimal anonymized data set necessary to replicate your study findings as either Supporting Information files or to a stable, public repository and provide us with the relevant URLs, DOIs, or accession numbers. For a list of acceptable repositories, please see <http://journals.plos.org/plosone/s/data-availability#loc-recommended-repositories>

We will update your Data Availability statement on your behalf to reflect the information you provide.

Response: Please see response to 3a above.

4. Please review your reference list to ensure that it is complete and correct. If you have cited papers that have been retracted, please include the rationale for doing so in the manuscript text, or remove these references and replace them with relevant current references. Any changes to the reference list should be mentioned in the rebuttal letter that accompanies your revised manuscript. If you need to cite a retracted article, indicate the article’s retracted status in the References list and also include a citation and full reference for the retraction notice.

Response: Thank you we have reviewed the reference list and made necessary changes

Reviewers' comments:
Reviewer's Responses to Questions

**Comments to the Author**
1. Is the manuscript technically sound, and do the data support the conclusions?
The manuscript must describe a technically sound piece of scientific research with data that supports the conclusions. Experiments must have been conducted rigorously, with appropriate controls, replication, and sample sizes. The conclusions must be drawn appropriately based on the data presented.

Reviewer #1: Yes

2. Has the statistical analysis been performed appropriately and rigorously?

Reviewer #1: Yes

3. Have the authors made all data underlying the findings in their manuscript fully available?
The [PLOS Data policy](http://www.plosone.org/static/policies.action#sharing) requires authors to make all data underlying the findings described in their manuscript fully available without restriction, with rare exception (please refer to the Data Availability Statement in the manuscript PDF file). The data should be provided as part of the manuscript or its supporting information, or deposited to a public repository. For example, in addition to summary statistics, the data points behind means, medians and variance measures should be available. If there are restrictions on publicly sharing data—e.g. participant privacy or use of data from a third party—those must be specified.

Reviewer #1: Yes

4. Is the manuscript presented in an intelligible fashion and written in standard English?
PLOS ONE does not copyedit accepted manuscripts, so the language in submitted articles must be clear, correct, and unambiguous. Any typographical or grammatical errors should be corrected at revision, so please note any specific errors here.

Reviewer #1: Yes

5. Review Comments to the Author
Please use the space provided to explain your answers to the questions above. You may also include additional comments for the author, including concerns about dual publication, research ethics, or publication ethics. (Please upload your review as an attachment if it exceeds 20,000 characters)

Reviewer #1: The authors have appropriately addressed an important research question in the manuscript.


Lines 76-77: Please rephrase the statements.

Response: Thank you, this has now been addressed. (Line: 77)

Lines 93-100: Have not been referenced

Response: Thank you, the reference for the same has been added.

Lines 146-155: More details could be provided on conduct & outcomes of the workshop

Response: Lines: 157 to 160 - Details on the conduct & outcomes of the workshop is now provided. This has enriched the content of the manuscript (Line: 157 to 160) . Thank you.

Line 169: What was done during the preparatory period of one year?

Response: During the preparatory period of one-year desk review, stakeholder cum expert workshop, module development, pilot training and evaluation methods were finalised. The same is also included in the article. (Lines: 174 to 175)

Lines 175-177: The statements need to be rephrased. More details need to be provided about the selection & timing of control group.

Response: Thank you the mentioned statements are rephrased and added details about the selection and timing of control group. (Lines: 179 – 182)

Line 185: Bilingual tool – development, translation & validation details to be mentioned

Response: The contents of the tool was based on the available literature and including different standardised scales. The same is also included in the manuscript. (Lines: 195-196 and 209-211)

Line 255-258: Written informed consent needs to be included

Response: This is included in the methods (Lines: 186 & 270). Thank you.

Table2: What does -worried about their concerns mean?

Response: Thank you for this important observation. We have now changed this to “Worried about Family members’ concerns”

Table 3: were the participants able to clearly define – feeling depressed, feeling anxious & having suicidal ideation?

Response: Yes, as the statements in the tool clearly defines it as well as the statements leading to it defines it. Also, as there were trained project staff during the tool administration the clarifications were provided for the participants while responding to those statements.

Table 4: Details about different traits & quality of life components to be provided in methods or provided as supplementary material

Response: This is provided in table 1 of the manuscript and also will be provided as supplementary material

6. PLOS authors have the option to publish the peer review history of their article ([what does this mean?](https://journals.plos.org/plosone/s/editorial-and-peer-review-process#loc-peer-review-history)). If published, this will include your full peer review and any attached files.
If you choose “no”, your identity will remain anonymous but your review may still be made public.
**Do you want your identity to be public for this peer review?** For information about this choice, including consent withdrawal, please see our [Privacy Policy](https://www.plos.org/privacy-policy).

Reviewer #1: No

Editors comments

Introduction:

1. Line 87: Authors mention tobacco use (35.6%), however the reference no. 5 does not have a link and the citation will not lead to appropriate reference. Pls verify.

Response: Thank you for noticing. The reference is now complete.

Methodology

1. Page 7. Line 143 to 145: We finally reviewed 77 articles to gather information regarding different modules used for youth specific Life skills training and available tools to assess the effectiveness of such life skills training program.

Needs to be mentioned in results section

Response: This is included in the results sections (Lines: 276 to 278). Thank you.

2. Table 1: Section 1, Interview information. The points are not clear, need to elaborate.

Response: The points under section 1 interview information is elaborated in Table 1

3. Pg 14. Line 216. The Data collection procedures documented in a written protocol can be provided as supplementary material for reference of readers.

Response: The written protocol on data collection procedures is provided as supplementary file

4. Pg. 15, Line 222 -223. Rephrasing required.

Response: Thank you. These sentences are rephrased in manuscript (Lines: 234 to 240)

Discussion:

1. Repetition can be avoided. Line 377 to 379 has been mentioned in earlier section also.

Response: Thank you for the observation. This is avoided in the discussion section. (Lines: 391to 393)

2. Pg 29. Line 440 to 449. It would be better to restrict the findings to present study.

Response: The above mentioned points relate to the strengths and limitations of the methodology adopted (and not a finding of this study) for the present study like control group getting trained eventually, contamination etc. (Lines: 454 – 4643). We feel that this is important to be retained. We request you to kindly consider retaining the same. Thank you.

References

1. Ensure links are working.

Response: Thank you for noticing this. The links are re-checked and the provided links are working

2. Ref 5, 7 and 22 needs to be as per the standard guidelines.

Response: The mentioned references are re-checked and made necessary changes as per the standard guidelines.
